# Building nurse education capacity in India: insights from a faculty development programme in Andhra Pradesh

**DOI:** 10.1186/1472-6955-12-8

**Published:** 2013-03-27

**Authors:** Catrin Evans, Rafath Razia, Elaine Cook

**Affiliations:** 1 School of Nursing, Midwifery and Physiotherapy, University of Nottingham, Queens Medical Centre, Nottingham NG7 2UH, U.K; 2 Government College of Nursing Hyderabad, Dr NTR University of Health Sciences, Andhra Pradesh, India

**Keywords:** India, Nursing, Faculty development, Andhra Pradesh, Education, Capacity development

## Abstract

**Background:**

India faces an acute shortage of nurses. Strategies to tackle the human resource crisis depend upon scaling up nursing education provision in a context where the social status and working conditions of nurses are highly variable. Several national and regional situation assessments have revealed significant concerns about educational governance, institutional and educator capacity, quality and standards. Improving educational capacity through nursing faculty development has been proposed as one of several strategies to address a complex health human resource situation. This paper describes and critically reflects upon the experience of one such faculty development programme in the state of Andhra Pradesh.

**Discussion:**

The faculty development programme involved a 2 year partnership between a UK university and 7 universities in Andhra Pradesh. It adopted a participatory approach and covered training and support in 4 areas: teaching, research/scholarship, leadership/management and clinical education. Senior hospital nurses were also invited to participate.

**Summary:**

The programme was evaluated positively and some changes to educational practice were reported. However, several obstacles to wider change were identified. At the programme level, there was a need for more intensive individual and institutional mentorship as well as involvement of Indian Centres of Excellence in Nursing to provide local (as well as international) expertise. At the organisational level, the participating Colleges reported heavy workloads, lack of control over working conditions, lack of control over the curriculum and poor infra-structure/resources as ongoing challenges. In the absence of wider educational reform in nursing and government commitment to the profession, faculty development programmes alone will have limited impact.

## Introduction

This paper provides a critical account of a nursing faculty development partnership that was implemented in the Indian State of Andhra Pradesh from 2009–2011. It has been written as a joint endeavour by representatives of the UK and Indian nurse educators who were involved.

We begin by contextualising the faculty development programme by providing an overview of the current challenges and opportunities facing nursing education in India – described by a recent Lancet article as ‘in crisis’ and facing near collapse in several poor but highly populous states [1]: 593. Improving educational capacity through nursing faculty development has been proposed as one of several strategies to address a complex health human resource situation [1]: 596. We then go on to describe and critically reflect upon our experience of one such faculty development programme and identify lessons for future consideration.

## Background

### The Indian context

India is undergoing a period of unprecedented social and economic change. Amongst its 1.2 billion population, economic growth has led to a rapidly expanding urban middle class. At the same time, a large proportion of the country’s population still reside in rural areas in conditions of economic hardship, low literacy and poor health. Increasing migration to the cities for work has created large urban slums lacking in basic amenities. This demographic situation means that the country faces the dual challenge of tackling diseases of poverty alongside an increasing incidence of chronic diseases more traditionally associated with westernised affluent lifestyles. The Indian health system is pluralistic, comprising public, private and voluntary sector facilities, of which the private sector is by far the largest provider. Since 2005, there has been enormous government investment into modernising and expanding India’s public healthcare system through the setting up of a new initiative - the “National Rural Healthcare Mission” [2]. Health system reform is constrained however by an acute shortage of health workers at every level [3]. In addition, poor health system governance (i.e. inadequate systems to monitor and regulate training institutions, professional practice and clinical standards within different settings) has been identified as a critical factor impeding efforts to improve quality and accountability, in both private and public sectors [1].

### Nursing in India: a profession in transition

India faces an acute shortage of nursing staff with an estimated deficit of 2 million [4]. In the public sector alone, an additional 140,000 staff nurses are required [5]. The nurse-population ratio is 1:2,500 compared with ratios of 1:150 to 1:200 in higher income nations [6]. The nurse-doctor ratio is also poor – at 0.5 nurses per doctor compared with 3 or 5 per doctor in the USA and UK respectively [1,6].

The development of nursing in India reflects the country’s history and complex socio-cultural composition. Traditionally, amongst Hindu and Muslim communities, the need for female nurses to work outside of the home (including at night), to touch strangers, to mix with men, and to deal with bodily fluids (considered polluting within Hindu and Muslim cosmology) has meant that until relatively recently, nursing was a stigmatised and low status profession [7]. During colonial times, British missionaries attempted to redefine and professionalise nursing as a respectable vocational career [8]. British mission hospitals established nursing schools and recruited poor women or widows from predominantly Christian communities, many from the southern Indian state of Kerala [9]. Kerala remains a major supplier of Indian nurses, although this is changing due to a shift in the desirability of nursing as a career that has come about because of increased opportunities for migration to the Middle East and further afield [10]. As in many other countries, nursing is now seen as a potentially lucrative career choice, a stepping stone to work overseas and towards greater social mobility for the entire family [11,12]. This has led to an influx of men into the profession and to a positive change in the social status of nurses [13]. Nonetheless, in India and throughout South Asia, the desire to avoid the stigma associated with basic nursing tasks forms a strong cultural backdrop to the way in which clinical nursing is valued and practised today [7,14-16].

Research evidence on nurses’ working conditions and job satisfaction in India is limited. However, reports indicate that nursing lacks clear career pathways and mechanisms for promotion; in-service training is rare (except in the best corporate hospitals); pay is low (especially in small private hospitals); and working conditions are often inadequate, lacking sufficient staff, equipment and infra-structure [17-19]. One study in New Delhi, found that nurse:patient ratios of 1:50 were the norm [13]. In the same study (which was based on over 150 interviews) nurses reported spending much of their time doing administrative, menial or unskilled work [7,13]. In a study of female health workers in Kolkata, more than 50% of respondents admitted experiencing sexual harassment at work [20]. Nurses in private hospitals in New Delhi recently staged a strike in protest of low pay and exploitative working conditions [19].

The nursing profession lacks strong strategic representation at key decision making forums at both State and National levels [18,21]. Nursing is governed through the national Indian Nursing Council (INC) and State level Nursing Councils (SNCs) [17] The INC advises the government on nursing matters, prescribes national nursing education syllabi and specifies minimum quality criteria for educational institutions. State Nursing Councils inspect and accredit training institutions, conduct examinations, monitor rules of professional conduct and maintain an active register. However, the legal authority of the INC is weak [17]. For example, a recent survey concluded that 61% of all nurse training institutions do not meet INC standards, but it is unable to take action as the institutions have nonetheless been accredited by the SNCs [3]. Nursing is also represented by a number of state and city based organisations, including the national Trained Nurses Association of India (TNAI). Greater nursing participation in health workforce policy making has been urgently recommended [1]. The INC is currently not a member of the International Council of Nursing.

### Nursing education in India

There are 2 main routes into nurse training in India. The majority of nurses undergo a 3 year diploma training in Schools of Nursing to become a General Nurse Midwife (GNM). A minority undertake a 4 year training in a College of Nursing (affiliated to a University) to obtain a BSc degree, referred to as BSN. Apart from the pre-registration programmes described above, University Colleges of Nursing also offer post-registration BSc courses and MSc courses. A national consortium of 5 universities came together in 2005 to start a collaborative nursing PhD programme [22].

In most public sector healthcare facilities, staff nurses are recruited from the GNM cadre (diploma-holders) only. Studies suggest that BSc graduates tend to seek clinical work in the private sector but often view this as a short- term strategy to gain requisite experience to enable overseas migration [13]. Post-registration BSc and MSc graduates are reported to move predominantly into educational positions in the public and private sectors [6]. Thus, as in many countries where clinical nursing carries a low status, academic qualifications are valued as a potential route out of clinical practice into higher status and better paid jobs in education [15].

Due to increasing demand for nurses nationally and internationally, India has witnessed a dramatic proliferation of nursing education institutions in recent years, although there is still an overall shortage. Over 88% of nurse education is now delivered in the private sector. There is also a geographical imbalance in nursing education, with most graduate and postgraduate education being delivered in the South. For example, the highly populous but poorer States in the North (e.g. Bihar, Madhya Pradesh, Rajasthan and Uttar Pradesh) account for only 9% of nursing schools in the country [1].

Several reports have highlighted significant problems in nursing education, emphasising that quality must not be sacrificed in the country’s current drive to scale up nurse training provision. Key issues are summarized below [1,3,6,10,18,21]:

• Inadequate educational monitoring and governance at State level (for example, sub-standard institutions continue to receive accreditation despite being unable to meet INC and University standards)

• Serious teaching staff shortages

• Poor physical infrastructure

• Poor educational infrastructure and resources, especially for clinical skills teaching

• Lack of continuing professional development for faculty

• Lack of promotion opportunities for faculty

• Over-cluttered curriculum

• Reliance on didactic teaching approaches

• Poor student living accommodation

• Poor links between clinical areas and educational institutions

• Inadequate clinical experiences (e.g. some placements have too many students; medical students take precedence over nursing students in practising key skills such as deliveries; nursing students may never get the opportunity to gain key clinical competencies)

Amidst the challenges, it is important to point out that there are, of course, also many Centres of Excellence in nursing education in India, but there is limited published material documenting their successes, systems and processes.

One commonly recommended strategy to improve nursing education is to recruit more faculty and to support existing faculty to develop their educational provision and practices [21]. Below, we report on one such initiative from the State of Andhra Pradesh.

## Discussion

### The Andhra Pradesh nursing faculty development programme

Andhra Pradesh is a large state on the south-east coast of India with a population of almost 76 million. The main language is Telugu. The capital city is Hyderabad. In the period between 2004–2008, the State Government approached an international non-governmental organisation (with a history of innovation in nursing education in South/Central Asia and east Africa) to assist nursing education. Following a number of needs assessments, a Nursing Faculty Development Programme (NFDP) was initiated in 2008 for faculty from 4 public and 2 autonomous nursing education institutions in Andhra Pradesh. The primary objective of the NFDP was to strengthen the capability and capacity of nursing faculty within the State. A Government College of Nursing in Hyderabad (GCNH) was selected to act as a nodal agency for the NFDP. The School of Nursing, Midwifery and Physiotherapy (SNMP), University of Nottingham (UK) won a tender to act as an international partner to the NFDP. The original plan was for the SNMP to work with the GCNH to provide some faculty development courses along a ‘training of trainers’ (ToT) model, so that subsequent faculty development programmes in the State would be delivered through the GCNH.

### Faculty development methodology

A review of previous literature on international partnerships indicated that the NFDP would need to adopt a collaborative approach in order to ensure that the inputs addressed common goals, aligned with local issues and were relevant to the national and local context [23-26]. The NFDP was based on a philosophy of mutual respect and adult learning [27]. A participatory approach was adopted for the entire curriculum development process so that each input ended with a formative evaluation and a collaborative planning process to shape the next input [28].

#### NFDP steps

The NFDP included the following steps:

1. Conference in Hyderabad to launch the programme and an initial participatory planning workshop for a core group of Andhra Pradesh faculty to identify and prioritise training and development issues

2. Delivery of a leadership workshop (for senior Andhra Pradesh faculty) by senior SNMP staff in Hyderabad

3. Delivery of 2 modules by SNMP faculty in Hyderabad

4. Visit of 6 Andhra Pradesh nursing faculty to SNMP, UK

5. Delivery of 2 further modules by SNMP faculty in Hyderabad

6. Formative evaluation

#### Needs assessment and planning

During an initial curriculum development workshop, a group of faculty from across Andhra Pradesh identified 4 main areas of input for the NFDP. These were:

1. Learning about new educational approaches (particularly experiential learning)

2. Being supported to develop their own careers through research, scholarship and publication

3. Strengthening skills in leadership and management

4. Learning about innovations in clinical education

These 4 domains were very similar to those covered in other documented faculty development programmes [23,26,29-31], and were developed into 4 distinct modules – see Table 1.

**Table 1:** NFDP modules

**Title**	**Aim**	**Learning outcomes**
Advanced and innovative methods in nursing education	To further develop educators’ knowledge, skills and confidence in delivering advanced and innovative teaching methods in order to enhance student learning	● Identify innovative teaching methods;
		● Understand relevant personal resource and environmental issues in ensuring systematic application in learning methods;
		● Explore the role of the teacher in facilitating learning by utilising different teaching methods;
		● Develop and apply advanced and innovative teaching methods in order to enhance student learning;
		● Explore different learning styles and strategies and how they relate to student learning;
		● Evaluate assessment strategies associated with innovative teaching and learning;
		● Examine the advanced and innovative methods of evaluating learning;
Developing and advancing scholarship	To build capacity in nursing scholarship for nursing faculty and propose strategies for improving scholarship in Andhra Pradesh	● Outline a systematic approach to scholarship in order to develop an action plan for future professional advancement;
		● Building knowledge and skills in the scholarship of nursing education, research and practice;
		● Develop advanced skills in writing scholarly papers and funding proposals;
		● Cultivate strategies that support and facilitate scholarship;
		● Progress skills in disseminating scholarly activities;
Developing effective leadership and management in nursing education and practice	To facilitate the development of effective leadership in nursing	● Identify the leadership challenges for nursing in Andhra Pradesh;
		● Analyse self utilising critical reflection in order to develop professionally as a leader;
		● Develop skills and approaches to management and leadership in nursing;
		● Apply effective leadership strategies;
		● Identify the need for and implement change to improve care quality and the educational experience for students;
		● Prepare a professional development plan to promote and evaluate self-progression;
Developing clinical learning	To facilitate clinical learning utilising evidence based practice to enhance the students’ learning experience	● Explore the concept of practice learning within the context of AP;
		● Develop and evaluate an internship programme to facilitate student learning;
		● Develop nursing practice using an evidence base focusing on hand washing;
		● Facilitate partnerships between clinical staff and the Nursing Teaching Faculty;
		● Utilise models and tools to facilitate changes in clinical practice and learning;
		● Facilitate the dissemination of the programme outcomes through scholarly activity;

The faculty were keen to receive updates on particular clinical topics (e.g. critical care). Given the wide variation of interests amongst the teachers however, it was agreed that, although important, the first phase of the NFDP would focus on the generic areas outlined above. The programme subsequently included sessions whereby faculty were encouraged to consider how they could access such updates in future.

From the outset, it was recognised that implementation of any educational innovations as a result of new learning would require support from the senior leadership within the 7 participating nursing institutions [32]. For this reason, a workshop on “*Strategic Leadership for the Advancement of Nursing Scholarship*” was held for senior Andhra Pradesh faculty (College Deans and Principals) to help them to reflect upon their own leadership styles and challenges and to create an institutional plan of action to support educational innovation.

Upon the advice of the Research Ethics Officer from the University of Nottingham, School of Nursing, Midwifery and Physiotherapy, the NFDP was deemed to be an educational development initiative rather than a research or evaluation study. A formal ethical approval process was not required therefore for the purposes of recording and disseminating project outcomes. However, in accordance with good practice, all participant and institutional information have been anonymised. During one of the programme inputs (the module on ‘Developing and Advancing Scholarship’), participants were encouraged to identify ways in which they could develop their own scholarship and publication strategies. Participants suggested that one immediate action would be to disseminate lessons learnt from the NFDP to the wider nursing community and the second author agreed to take this forward by contributing to a paper.

#### Programme delivery

The inputs were delivered over a 2 year period (2009–2011). Each module ranged from 7–10 working days and resulted in a certificate of attendance. Each module concluded with the participants developing a detailed but realistic action plan for taking forward relevant learning. Progress with the action plans were then reviewed in the next module.

In total, 25 faculty members attended the modules (including 12 senior faculty). Six senior clinical staff also attended the leadership/management and clinical learning modules.

In addition to the modules, a visit of 6 senior Andhra Pradesh faculty to the SNMP in the UK was also conducted. The aim was to provide the opportunity to explore nurse education and practice outside India in order to consider new ways of working and, particularly, to identify the role of collaborative working relationships between education and clinical practice. This visit also provided time for reflection on the NFDP programme and future planning for longer term sustainability.

Alongside the educational development, the NFDP included funds to upgrade some facilities at GCNH, e.g. purchase of new skills equipment, provision of ten computers with internet access, provision of printing facilities for students and the purchase of books for the library.

### Evaluation and reflections on the nursing faculty development programme

Formative participatory evaluations were conducted by the SNMP at the end of each module and at the end of the programme. Participants filled in a brief module evaluation questionnaire and group discussions were held to explore participants’ and facilitators’ views of the module/programme delivery, impact on education and practice and prospects for longer term change. Key issues that emerged from the evaluations are reported below.

#### Developing innovations in teaching and learning

Overall, the NFDP delivery was evaluated extremely positively both in terms of content and the experiential/student-centred educational approaches adopted by the facilitators. These approaches were initially very challenging for the participants whose previous educational experiences had been predominantly didactic in nature – and this shaped their expectations of the NFDP. Using experiential approaches initially moved many of the participants out of their own comfort zones as the SNMP facilitators used a wide range of techniques to ensure that all members of the group participated.

A related issue was that in all the modules, participants were expected to reflect upon, and share, their existing knowledge and experience in order to identify their own needs for future personal development, and to consider how their institutional processes or practices could change to improve educational quality. Again, this was challenging at times. For some participants, reflection, problem identification, goal setting and action planning were somewhat alien concepts in a context where marked occupational hierarchies as well as rigid bureaucratic processes create a (realistic) sense that change is difficult, and that individual initiative may not always be welcome.

Facilitating the participants’ learning was also demanding at times for the SNMP faculty who were challenged to adapt their teaching style and content. Both participants and facilitators agreed that the relevance of some of the module content would have been improved if the SNMP staff had had greater experience of Indian higher education and nursing contexts. This important issue is addressed further below.

It had originally been envisaged that the participants in the NFDP would complete all of the modules and would thus get used to different teaching styles and techniques over time. In reality however, although some participants completed all 4 modules, there were also different participants each time which affected the group cohesion and learning process. Nonetheless, over time many NFDP participants noted that their confidence and motivation had improved and that they were applying new skills with respect to teaching - particularly in structuring lectures and group work more effectively, evaluating student learning, using new tools, incorporating NFDP module content into their own teaching and in making learning more enjoyable (e.g. by using humour or interactive techniques).

In spite of the challenges, both groups stated that the programme had provided a tremendous opportunity for cross-cultural learning and for creating a deeper understanding of nursing in a global context.

#### Clinical education, status issues and the theory- practice gap

Although the clinical learning module was evaluated positively, many issues were raised which resonate with the existing literature on nursing in India and which created real challenges for innovation. Due to their critical importance, the key issues that emerged are outlined below.

Clinical teaching was seen as the responsibility of the faculty who were expected to visit the students on the wards every day (and then return to the College to carry on with classroom teaching). However, heavy workloads meant that their time and availability to students was sometimes limited yet little instruction took place in their absence. Staff nurses did not see it as their role to support students’ learning and they were usually busy with their own tasks. Equipment for teaching clinical procedures was not provided to the students from ward stock. Rather, faculty needed to bring their own supplies with them (as is common in times of scarcity, staff nurses tended to lock precious equipment away in case of breakage or loss). In addition, for student cohorts studying to BSc and MSc levels, the fact that staff nurses predominantly had a diploma qualification created status ambiguities in terms of the staff nurses’ deemed ability to support students studying at a higher educational level than themselves.

Lack of resources, capacity and infrastructure also created a deep theory-practice gap in the students’ learning. For example, students would be taught about processes (e.g. nursing assessments, care planning or particular clinical procedures) that had no relation to the realities of practice and that they had never witnessed. Faculty readily admitted that they themselves lacked the clinical skills to teach some of the prescribed procedures. These anomalies had to be perpetuated however due to the need for faculty to follow the prescribed INC curriculum and for students to pass exams based on that curriculum. In some cases, even where opportunities existed (e.g. to conduct a delivery), medical students reportedly took precedence over nursing students. The unregulated proliferation of private Nursing Schools was also creating additional pressures for clinical placements. For example, in some clinical areas there could be up to 50 students, all coming from different institutions, yet there was no evidence of coordination among these institutions.

The SNMP had deliberately suggested including senior clinical staff in the leadership/management and clinical learning modules in the hope that this might open up space for dialogue to consider ways in which faculty and staff nurses could work together more closely to support students’ learning in practice. This required careful facilitation and sensitivity to occupational hierarchies – for example, initially one of the senior clinical nurses remarked that “*educators think that we do not know anything*”. In time, constructive dialogue was achieved and many suggestions were forthcoming as to how education and practice could work in partnership. At the time of writing however, it is unclear as to whether any changes have taken place.

#### Organisational/institutional context and nursing faculty development

As noted above, the institutional context in which the NFDP took place created real challenges for the possibility of educational development. The new facilities (e.g. computers) at GCNH were reportedly well used and had improved the educational experience for the students. However, other participating institutions reported a similar need to upgrade their facilities. A new building for the GCNH had also been promised – symbolising a real commitment by the state government to nursing development - though this has not yet been realised.

Other innovations were more difficult to achieve. For example, the participants reported feeling relatively limited in their scope for innovation as the nursing curriculum (content, time allocation, teaching and assessment strategies) was prescribed in great detail by the INC, leaving little room for flexibility.

In addition, faculty from 6 out of 7 of the participating institutions reported excessive workloads and staff shortages as severe obstacles to undertaking potentially time/labour intensive innovations in educational practice. For example, during the NFDP period, the GCNH had its MSc intake doubled with no additional staff allocation. A lack of control over working conditions and pressure to meet immediate teaching requirements meant that few participants or institutions reported undertaking any significant educational innovations as a result of the programme.

Another challenge was that there was no mechanism within the NFDP for participating institutions to meet each other or to receive on-going mentorship or support in the time-periods between the modules. This meant that any momentum and enthusiasm built up during a module understandably faltered in the intervening months. Moreover, there was no mechanism within the NFDP for the participating institutions to network with Indian Centres of Excellence in Nursing Education. Although the input from the UK SNMP was appreciated, the vastly differing contexts of healthcare, nursing and the nursing curriculum between the 2 countries created real challenges for the SNMP facilitators to work in partnership with the Andhra Pradesh faculty to identify locally relevant and realistic strategies for change.

### Summary

The NFDP has brought welcome resources and attention to nursing education in the state of Andhra Pradesh. Amongst faculty, it has achieved an awareness of new educational approaches and enthusiasm for on-going professional development. There have been some innovations made to day to day teaching practice. More significant changes have not been tackled however. The originally conceived ToT model of nursing faculty development seems doubtful as the future trainers have not yet themselves had the opportunities to put new approaches to nursing education into practice, thereby limiting the existing programme to the development of greater theoretical rather than experiential expertise. The deeper, structural problems affecting nurse education quality remain relatively unresolved.

Based on the valuable experience of the NFDP, we conclude this paper with some suggestions for future nursing faculty development initiatives in the Indian context.

First, whilst it is beyond the scope of a faculty development initiative to address national or state level policy, it is clear that educational initiatives alone will have a limited impact in the absence of work to review the nursing curriculum and regulation of nurse training institutions. Our experience shows that the current nursing curriculum is in need of review in order to better equip nurses to manage (and try to improve) the conditions of practice that they encounter, and to provide faculty with the autonomy and motivation to innovate.

Second, enlisting an international partner to support nursing faculty development undoubtedly provides a different perspective on nursing education and a different skill set that can be extremely valuable. Nonetheless, we would suggest that an Indian partner (drawn from recognised Centres of Excellence) should also be included as a key partner in the training team to build up local expertise, to enhance the prospects for longer term sustainability and to ground the module/programme content in the realities of the local context.

Third, it is well recognised in the literature that faculty development works best when faculty are supported in the long term by a system of mentorship to enhance personal development [33,34]. Likewise, mentorship can also be valuable at an institutional level whereby one Nursing School/College recognised for excellence provides on-going support to another [32,35,36]. We suggest that future faculty development initiatives include both forms of mentorship. This could, for example, consist of periodic visits from an international partner, coupled with more regular and intensive support from an Indian Centre of Excellence.

Finally, within the existing (relatively limited) literature on nursing education in India, there is a noticeable paucity of research on the student experience and on the views or practices of clinical staff in terms of their educational role. In order to base future faculty development initiatives on locally-relevant evidence, additional research on nursing education (particularly clinical learning) is required.

## Abbreviations

ANM: Auxiliary Nurse Midwife; GCNH: Government College of Nursing Hyderabad; GNM: General Nurse Midwife; INC: Indian Nursing Council; NFDP: Nursing Faculty Development Programme; NRHM: National Rural Health Mission; SNC: State Nursing Council; SNMP: School of Nursing, Midwifery and Physiotherapy; ToT: Training of trainers.

## Competing interests

The authors declared that they have no competing interest.

## Authors’ contributions

CE, EC and RR conceptualised the paper. CE and EC wrote the first draft. RR made modifications. CE wrote the final draft. All authors read and approved the final manuscript.

## Authors’ information

CE is a Lecturer in International Health; EC is Associate Professor and Head of the Division of Nursing. Both work at the School of Nursing, Midwifery and Physiotherapy at the University of Nottingham, UK.

RR is Principal of the Government College of Nursing Hyderabad and Director of Nursing in Andhra Pradesh, India.

The views expressed in this paper represent those of the authors alone.

## Pre-publication history

The pre-publication history for this paper can be accessed here:

http://www.biomedcentral.com/1472-6955/12/8/prepub
